# Selective Laser Trabeculoplasty Compared to Medication for Open‐Angle Glaucoma Patients: A Systematic Review and Meta‐Analysis

**DOI:** 10.1155/joph/9102711

**Published:** 2026-01-22

**Authors:** Samantha So, Teng Qing Wang, Amardeep Thind, Cindy M. L. Hutnik, Monali S. Malvankar-Mehta

**Affiliations:** ^1^ Department of Epidemiology and Biostatistics, Schulich School of Medicine and Dentistry, The University of Western Ontario, London, Ontario, Canada, uwo.ca; ^2^ Department of Medicine, Michael G. Degroote School of Medicine, McMaster University, Hamilton, Ontario, Canada, mcmaster.ca; ^3^ Department of Ophthalmology, Schulich School of Medicine and Dentistry, The University of Western Ontario, London, Ontario, Canada, uwo.ca

## Abstract

**Background:**

An alternative glaucoma treatment is selective laser trabeculoplasty (SLT) that can effectively reduce intraocular pressure (IOP) and decrease the burden of glaucoma management. With newly published randomized controlled trials (RCTs) comparing SLT and medication, an updated systematic review and meta‐analysis was needed.

**Methods:**

A literature search of RCTs comparing SLT and medication in open‐angle glaucoma patients was conducted until January 12, 2024 in CINAHL, Cochrane Library, EMBASE, MEDLINE, Web of Science, ClinicalTrials.gov, and Dissertations and Theses databases. Cochrane Risk of Bias 2 (ROB2) was used to assess the quality of the included RCTs.

**Results:**

The included 16 RCTs comprised 2412 patients. At 26 weeks, the combination treatment of SLT and medication had a significant IOP reduction (SMD = 0.78, 95% CI = [0.56, 1.01], *p* < 0.01, *I* ^2^ = 0.00%). At 52 weeks or longer, both the SLT group (SMD = 1.91, 95% CI = [1.55, *I* ^2^ = 73.99%) and the medication group (SMD = 1.70, 95% CI = [1.01, 2.38], *p* < 0.01, *I* ^2^ = 92.87%) had significant IOP reduction. At 6 months, the combination treatment significantly reduced the mean number of medications used by 0.78 (SMD = 0.78, 95% CI = [0.56, 1.01], *p* < 0.01, *I* ^2^ = 0.00%) where the medication treatment had no significant decrease (SMD = −0.01, 95% CI = [−0.23, 0.21], *p* = 0.93, *I* ^2^ = 0.00%). Quality of life between participants in the SLT and medication groups was found to be similar.

**Conclusion:**

Long‐term IOP reduction was greater in the SLT treatment compared to medication. SLT treatment significantly reduced the number of medications used by participants whereas medication group had no significant change.

## 1. Introduction

Glaucoma is an eye disease causing damage to retinal ganglion cells of the eye and is one of the most important causes of irreversible blindness in the world [1, 2]. The most common form of glaucoma, primary open‐angle glaucoma (POAG), is differentiated by an open anterior chamber angle and increased resistance to drainage in the trabecular meshwork that results in ONH cupping and visual field loss [3–5]. It is estimated to affect 68.56 million people as of 2021 [6], but there are potentially an estimated 43.78 million cases of undetected POAG [7].

Currently, the main target of treatment in glaucoma is reducing intraocular pressure (IOP), as it seems to be one of the only modifiable risks that can slow or stop disease progression [8–10]. The initial treatment for IOP reduction is often topical medication [11], as it has seen widespread historical usage for its known efficacy [2, 12]. However, most topical medications are known to have both ocular and systemic side effects, which in addition to its high cost, can be detrimental to patient adherence to treatment. Furthermore, medication adherence can be especially difficult for patients living in regions with limited access to tertiary centers or pharmacies where their topical glaucoma medication prescriptions can be refilled easily [13].

Selective laser trabeculoplasty (SLT) is another form of IOP‐lowering treatment. Institutions have begun adopting laser trabeculoplasty as an alternate form of first‐line treatment either alone or in conjunction with topical medications [11], and one survey of Canadian ophthalmologists found that 17.7% of their sample uses SLT as first‐line treatment [14]. However, a majority of clinicians still prefer to use medication alone or medication combined with SLT as first‐line treatment [14].

One of the most recent systematic reviews and meta‐analyses comparing SLT and medication for OAG was published in 2024 by Chavez et al. They searched four databases (PubMed, Embase, Cochrane Library, and Web of Science) until January 2023 with no language limitations. Their systematic database search resulted in 14 randomized controlled trials (RCTs) and 1706 patients. There were two treatment groups in their analysis: SLT treatment and medication treatment. The follow‐up range of the RCTs was between 6 months and 6 years. The medication treatment varied between RCTs with most using stepped medication regimens. Most studies used 360° SLTs. The outcomes collected included IOP, IOP reduction, number of medications, success rate of IOP control, quality of life, and adverse events. Their meta‐analysis of the outcome of successful ≥ 20% IOP reduction found that a greater proportion of patients achieved this when they received the medical treatment compared with SLT. Chavez et al. also discovered that SLT provided comparable IOP, quality of life, and visual field preservation to medication treatment for most follow‐up periods. Their findings support SLT as a safe and effective OAG treatment compared with medication treatments [15].

Another recent review by Zhang et al. compared SLT and argon laser trabeculoplasty with drug therapy. This review included 18 studies, but only 12 of these were pertaining to SLT, while the other studies focused on argon laser trabeculoplasty. Although the total sample size of their review was 2024 patients, only 1545 of these patients were from studies pertaining to SLT, whereas the remainder were patients undergoing argon laser trabeculoplasty. They included two treatment arms: laser trabeculoplasty with SLT and argon laser subgroups as well as medication‐only treatment. The outcomes examined in this review included success rate of IOP control, average change in IOP, change in number of medications use, QoL, and adverse events. Their meta‐analysis found no significant difference in the rate of successful IOP reduction (defined in most studies as IOP below 21 mmHg) as well as the average change in IOP between laser trabeculoplasty and medical management. They also reported a significantly greater incidence of adverse events from laser trabeculoplasty compared with medication only and that laser trabeculoplasty significantly reduce the number of medications needed [16].

Numerous RCTs have been published since the findings of the previous review were synthesized. Based on the current body of evidence, this systematic review and meta‐analysis aims to compare the effects of SLT‐related therapy versus medication alone for patients with OAG at different time points in the treatment process using subgroup analyses. This systematic review and meta‐analysis could aid clinicians and guideline‐makers in creating updated recommendations for the suitability of SLT as first‐line treatment of OAG.

## 2. Methods

This review was registered on PROSPERO (CRD42024505860). The Preferred Reporting Items for Systematic Reviews and Meta‐analysis guidelines was followed when conducting this review.

### 2.1. Search Strategy and Study Selection

The literature search was conducted until January 12, 2024, with a limit to human studies, in the following five databases: CINAHL, Cochrane Library, EMBASE, MEDLINE, and Web of Science. The gray literature search was conducted in ClinicalTrials.gov, and Dissertations and Theses databases. The search strategy was composed using Boolean algebra to capture publications containing the terms, OAG (open angle glaucoma^∗^ or primary open angle glaucoma^∗^ or POAG) and SLT (laser trabeculoplasty or SLT or Yag laser trabeculoplasty or SLT or Nd YAG laser trabeculoplasty). All citations resulting from the search strategy were imported into Covidence [17], an online systematic review screening tool, to remove article duplicates and to screen articles. The two reviewers (S.S. and T.Q.W.) independently screened the records based on the inclusion and exclusion criteria. The two independent levels of screenings were: (1) title and abstract and (2) full‐text reviews. The level one screening question was: “Is it an RCT that explores the impact of SLT in open‐angle glaucoma adult patients?” The level two screening question was: “Does the RCT study the impact of SLT‐related treatment compared to medications in open‐angle glaucoma adult patients?” Through discussion between the reviewers, a consensus was reached to resolve conflicts at both levels of screening.

### 2.2. Inclusion and Exclusion Criteria

The inclusion criteria for this study were: (1) the study design was an RCT; (2) the study recruited adult patients diagnosed with OAG; and (3) the study intervention included SLT‐related therapy. The exclusion criteria were: (1) the study recruited children or adolescent patients; (2) the adult patients recruited were diagnosed with normotensive glaucoma or ACG; (3) the study intervention combined SLT therapy with other eye treatment (e.g., cataract removal); and (4) the gray literature was an incomplete study or lacked reported information. The SLT‐related therapy describes interventions that include SLT treatment and is separated into two groups: SLT‐only group and SLT + medication group. The SLT‐only group was defined as patients who received only SLT therapy as the start trial intervention. The SLT + medication group was defined as patients who received SLT and glaucoma medication simultaneously as the start trial intervention.

### 2.3. Quality Assessment

The Cochrane ROB2 was employed to assess the methodological bias of the included RCTs. The ROB2 measures risk of bias based on five factors: (1) randomization process, (2) deviations from intended interventions, (3) missing outcome data, (4) measurement of outcome, and (5) selection of reported results. In each domain, there are five response options (yes, probably yes, probably no, no, and no information) for the signaling questions to determine the level of bias. Each domain has an algorithm figure that helps to determine the suggested judgment for the risk of bias based on the answers to the signaling questions.

The overall bias of the study is determined as follows. If all domains have low risk of bias and no high risk of bias for any other domain, then the overall risk of bias is “low.” If there are some concerns for at least one domain, then the overall risk of bias is “some concern.” If at least one of the risks of bias domain is high or there are some concerns for multiple domains, then the overall risk of bias for the study is “high.” [18].

### 2.4. Data Extraction

Independent data collection was conducted and was compared manually by S.S. and T.Q.W. to extract the following information: study year, location, study population, and outcome data, including IOP reduction, the mean number of medications needed, the success rate of IOP control, quality of life (QoL) outcomes, and adverse ocular events (AOEs). The standardized mean difference (SMD) is the effect measure for the two outcomes: IOP reduction and the mean number of medications needed. If there were any missing data in a study, SS has reached out to the corresponding author of the study to acquire missing data.

### 2.5. Statistical Methods and Data Synthesis

Qualitative and quantitative analyses were performed using the included RCTs. The statistical heterogeneity was assessed using the I2 statistic with a value greater than 50% implying significant heterogeneity. Due to the heterogeneity of the studies, the random‐effects model was used for the meta‐analysis using a 95% CI for interval estimate. Subgroup analyses were conducted including “SLT versus medication” and “SLT + medication versus medication.” Additional analyses were performed by grouping RCTs based on time to follow‐up in weeks. For continuous outcomes, SMDs were calculated. Publication bias was evaluated using funnel plots. All statistical analyses were performed using STATA v.14.0.

## 3. Results

### 3.1. Search Results

**Figure 1:**
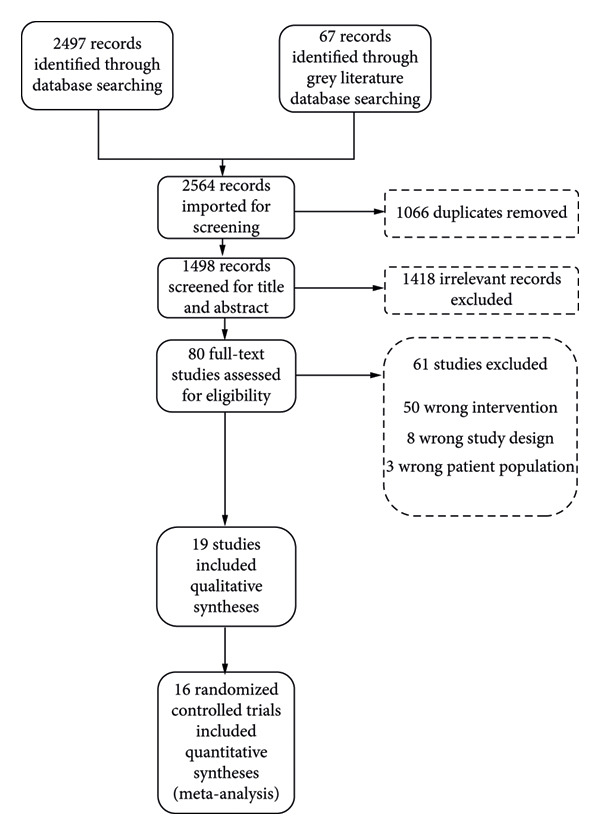
PRISMA diagram.

The 2497 published and 67 gray literature records were gathered from the database searches. After importing into Covidence, 1066 duplicates were removed. The systematic review screening process is detailed in a Preferred Reporting Items for Systematic Reviews and Meta‐Analysis (PRISMA) flow diagram (Figure 1) [19]. There were 19 RCTs that met the inclusion criteria with three of them being post hoc analyses [20–22] using the LiGHT Trial data. A total of 2412 patients from 16 RCTs were included in the systematic review and meta‐analyses [23–37].

### 3.2. Study Characteristics

This systematic review is composed of 2412 patients from Belgium [25, 38], China [35, 36], England [32, 33], Hong Kong [30, 31], Tanzania [34], Thailand [29], Turkey [37], the United Kingdom [26, 27], the United States [28], and international collaboration of different countries [23, 24]. The mean age and baseline IOP range from 51.90 to 70.28 years old and 15.2 to 29.3 mmHg, respectively. For medication treatment, most RCTs offered solely prostaglandin analog (PGA) [23, 24, 29, 32, 33, 35]. Philippin et al. used a PGA implant of bimatoprost for medication therapy, while all other studies had self‐administered eye drops interventions. There were two studies that only used beta‐blockers, specifically timolol, as the eye drop intervention [34, 37]. However, some studies did not have strict medication therapy, so patients used PGAs, carbonic anhydrase inhibitors (CAIs), alpha‐adrenergic agonists (AAs), or beta‐blockers therapies (BBs) [25–28, 30, 31, 36, 38]. For the SLT treatment, most RCTs performed the 360° SLT. The length of follow‐up for the included studies was between 6 weeks and 6 years. Characteristics of the included studies are detailed in Table 1. Based on the risk of bias assessments, there were one study with high RoB [24], 11 studies with some concern regarding RoB [23, 26–28, 30–35, 37], and four studies with low RoB [25, 29, 36, 38] (Figure 2). For any studies that did not perform SLT treatment blinding, they were graded with some concerns for the bias in measurement of the outcome (domain 4). Table 2 summarizes the significant results in IOP reduction, number of medication, and successful IOP control.

**Table 1:** Characteristics of included studies.

Study	Year	Study location	Follow‐up time (months)	People (*n*)	SLT group size (*n*)	Eyes (*N*)	Age (mean)	Female	Mean baseline IOP (mmHg)	Diagnosis	Prior glaucoma medication (weeks)
Ang et al.	2020	International	24	141	73	141	63.8	47.90%	20.1	POAG	Naive
Christie et al.	2023	Denmark, France, Poland, Russia, Singapore, Spain, Thailand, and the United States	12	138	138	276	60	46.53%	24.42	OAG OHT	6‐week washout period
De Keyser et al.	2017	Belgium	12	125	64	125	70.288	50.40%	23.07	OAG OHT	Keep prior medications
De Keyser et al.	2018	Belgium	18	125	67	244	70.16	50.82%	23.13	POAG OHT	N/A
Garg et al.	2019	Study used [27] baseline data.									
Garg et al.	2021	Study used [27] baseline data.									
Gazzard et al.	2019	United Kingdom	36	626	314	1072	63.0	44.71%	24.45	POAG OHT	Naive
Gazzard et al.	2023	United Kingdom	72	537	270	930	63.0	44.71%	24.45	POAG OHT	Naive
Katz et al.	2012	United States	9	54	30	100	N/A	59.42%	24.78	POAG OHT	4‐week washout period
Kiddee et al.	2017	Thailand	6 weeks, 8 weeks	30	16	30	N/A	N/A	21.48	POAG	4‐week washout period
Lai et al.	2004	Hong Kong	60	29	29	58	51.90	55.17%	26.50	POAG OHT	Naive
Lee et al.	2014	Hong Kong	6	41	22	22	66.2	46.46%	15.20	POAG	Keep prior medications
Nagar et al.	2005	England	12	167	128	167	63	53.89%	29.3	OAG OHT	5‐week washout period
Nagar et al.	2008	England	4–6	40	20	40	66.4	48.00%	24.45	OHT POAG	Naive
Philippin et al.	2021	Tanzania	12	201	101	382	66.3	41.29%	26.7	OAG	4‐week washout period
Shi et al.	2023	China	3	40	21	40	50.7	35.00%	20.685	POAG OHT	Naive
Tan et al.	2015	China	6	78	39	156	55.5	50%	20.76	POAG	No washout period
Tufan et al.	2017	Turkey	6	40	40	80	53.9	52.50%	16.8675	POAG	No washout period
Wright et al.	2020	Study used [27] baseline data.									

*Note:* F, female; M, male.

Abbreviations: IOP, intraocular pressure; LiGHT, laser in glaucoma and ocular hypertension trial; N/A, not applicable; OAG, open‐angle glaucoma; OHT, ocular hypertension; POAG, primary open‐angle glaucoma; SLT, selective laser trabeculoplasty.

Figure 2Risk of bias diagram (a) and summary (b).

**Figure:**
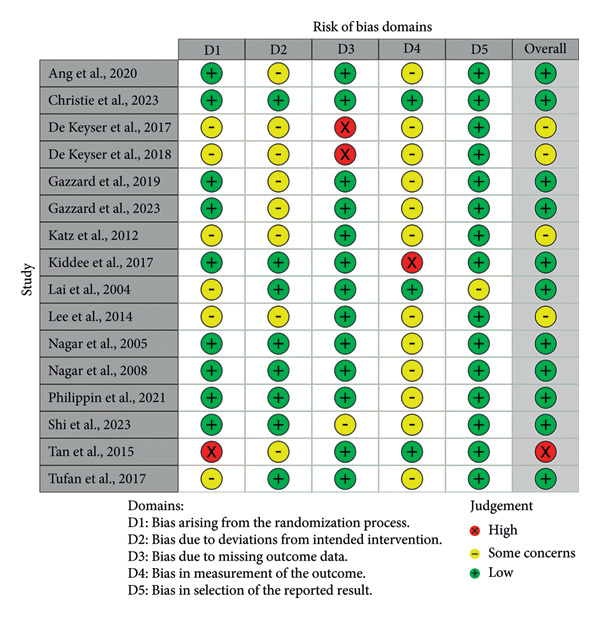
(a)

**Figure:**
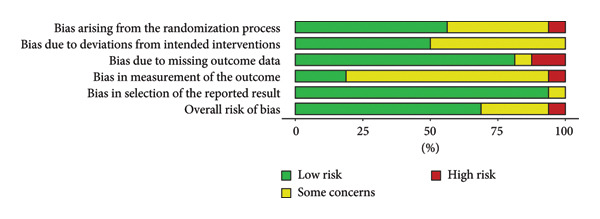
(b)

**Table 2:** Summary of IOP and number of medication analyses.

Study	Year	IOP reduction	Number of medications	Successful IOP control
Ang et al.	2020	N/A	N/A	‡
Christie et al.	2023	‡	N/A	N/A
De Keyser et al.	2017	†	∗	N/A
De Keyser et al.	2018	‡	∗	N/A
Gazzard et al.	2019	†	N/A	N/A
Gazzard et al.	2023	†	N/A	†
Katz et al.	2012	†	N/A	†
Kiddee et al.	2017	†	N/A	N/A
Lai et al.	2004	†	∗	N/A
Lee et al.	2014	∗	∗	N/A
Nagar et al.	2005	N/A	N/A	†
Nagar et al.	2008	†	N/A	†
Philippin et al.	2021	‡	N/A	‡
Shi et al.	2023	†	N/A	N/A
Tan et al.	2015	∗	∗	N/A
Tufan et al.	2017	†	N/A	N/A

^∗^SLT‐related treatment was significantly better than medication‐only treatment.

^‡^Medication‐only treatment was significantly better than SLT‐related treatment.

^†^No significant difference between interventions.

### 3.3. Primary Outcome: IOP Reduction

There was a total of 10 RCTs for SLT treatment, 13 RCTs for medication treatment, and three RCTs for the combination treatment of SLT and medication. A subgroup analysis based on follow‐up time was conducted to explore the difference in IOP reduction between different time points. These analyses are especially important in addressing the literature gap to assess the short‐ and long‐term impact of SLT on IOP reduction. For the SLT group (Figure 3(a)), the meta‐analysis revealed a significant pooled IOP reduction for the following follow‐up lengths: 1 week (SMD = 0.68, 95% CI = [0.13, 1.22], *p* = 0.02, *I* ^2^ = 72.07%), 8 weeks (SMD = 1.50, 95% CI = [0.50, 2.51], *p* < 0.01, *I* ^2^ = 95.86%), 24–26 weeks (SMD = 1.41, 95% CI = [0.63, 2.19], *p* < 0.01, *I* ^2^ = 94.05%), and 52 weeks or longer (SMD = 1.91, 95% CI = [1.55, 2.27], *p* = 0.01, *I* ^2^ = 73.99%). For the medication group (Figure 3(c)), the significant decrease in pooled IOP was found for 1 week (SMD = 0.90, 95% CI = [0.13, 1.67], *p* = 0.02, I2 = 84.61%), 4 weeks (SMD = 1.43, 95% CI = [0.10, 2.76], *p* = 0.04, *I* ^2^ = 96.97%; Figure 3(c)), 24–26 weeks (SMD = 1.00, 95% CI = [0.27, 1.73], *p* = 0.01, *I* ^2^ = 96.87%; Figure 3(c)), and 52 weeks or longer (SMD: 1.70, 95% CI = [1.01, 2.38], *p* < 0.01, *I* ^2^ = 92.87%; Figure 3(c)). The pooled analysis for the combination treatment reported a significant pooled IOP reduction for week 26 (SMD = 0.78, 95% CI = [0.56, 1.01], *p* < 0.01, *I* ^2^ = 0.00%; Figure 3(e)). The funnel plot for the SLT group and the medication group showed a large, mostly symmetric spread of the included RCTs, which may indicate some degree of publication bias (Figures 3(b) and 3(d)). For the combination therapy, there seems to be no evidence of asymmetry (Figure 3(f)).

Figure 3(a) Forest plot for intraocular pressure reduction in SLT‐only treatment group. (b) Funnel plot for intraocular pressure reduction in SLT‐only treatment group. (c) Forest plot for intraocular pressure reduction in medication‐only treatment group. (d) Funnel plot for intraocular pressure reduction in medication‐only treatment group. (e) Forest plot for intraocular pressure reduction in the combination treatment group at week 26. (f) Funnel plot for intraocular pressure reduction in the combination treatment group at week 26. (g) Forest plot for number of medications in the medication‐only treatment group. (h) Funnel plot for number of medications in the medication‐only treatment group. (i) Forest plot for number of medications in the combination treatment group. (j) Funnel plot for number of medications in the combination treatment group.

**Figure:**
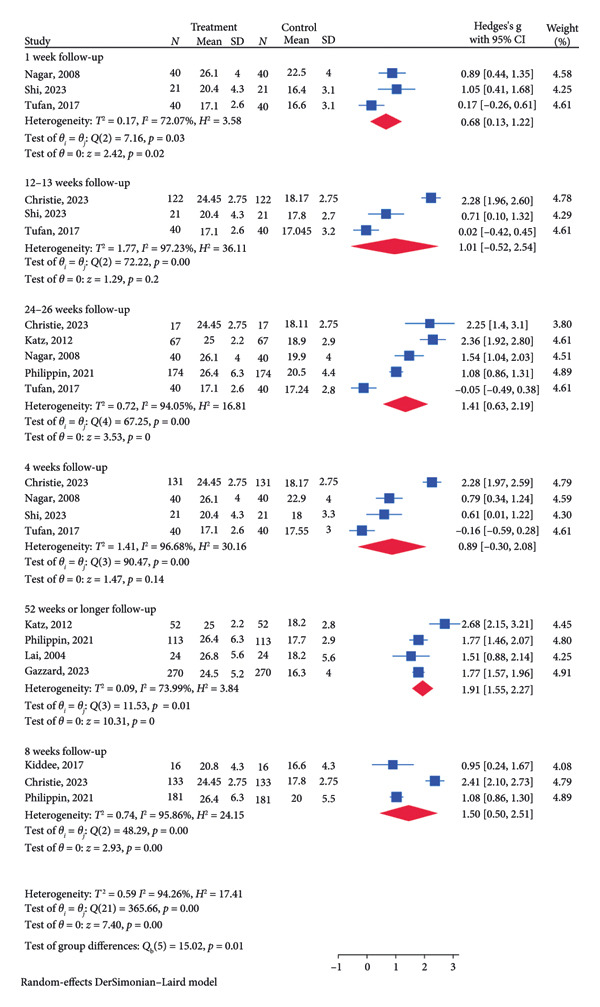
(a)

**Figure:**
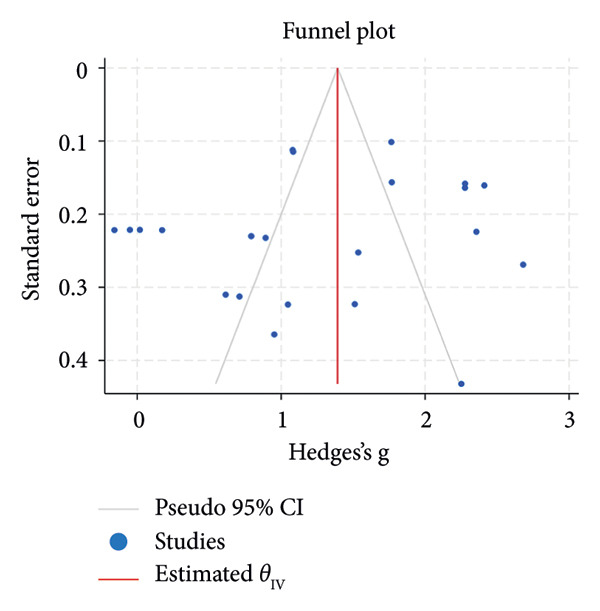
(b)

**Figure:**
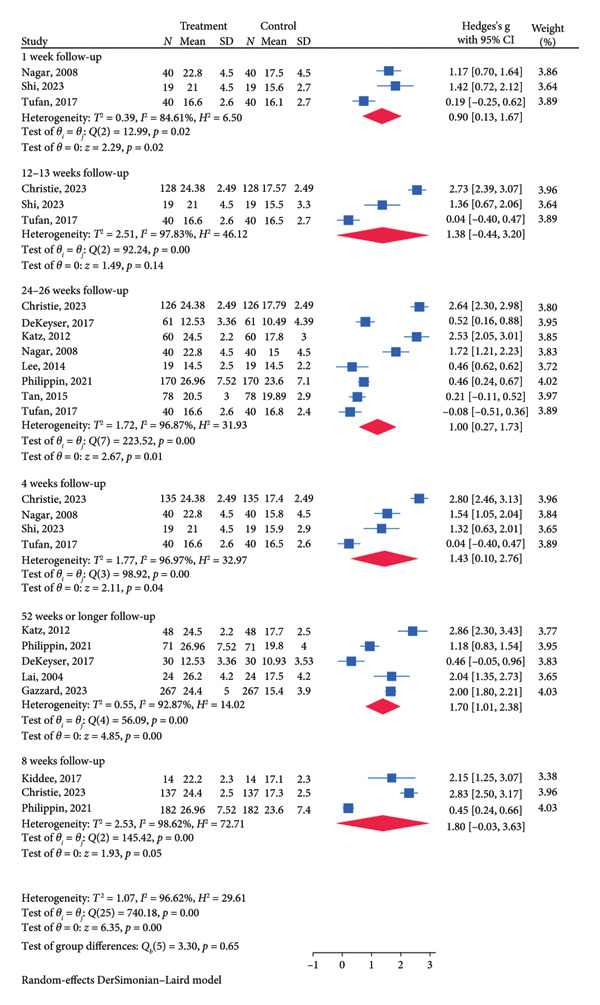
(c)

**Figure:**
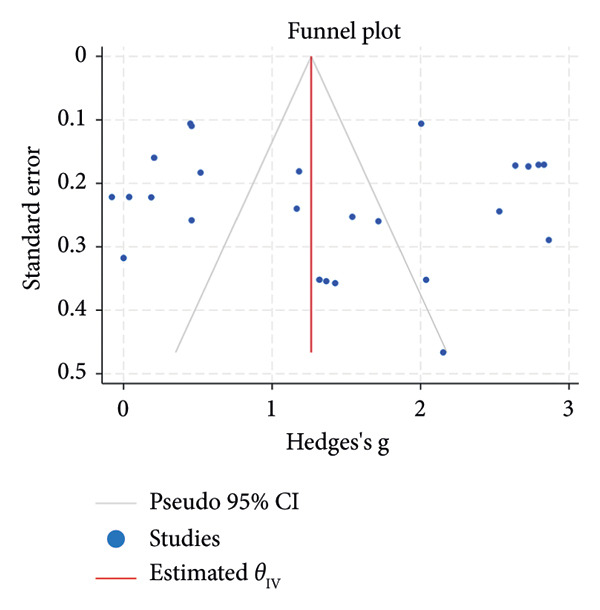
(d)

**Figure:**
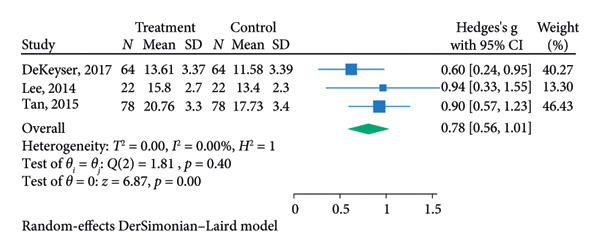
(e)

**Figure:**
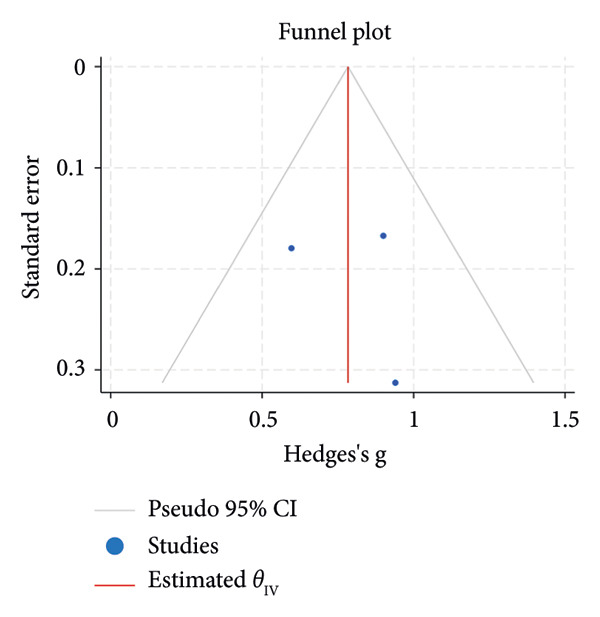
(f)

**Figure:**
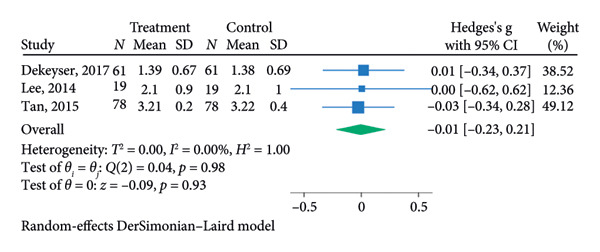
(g)

**Figure:**
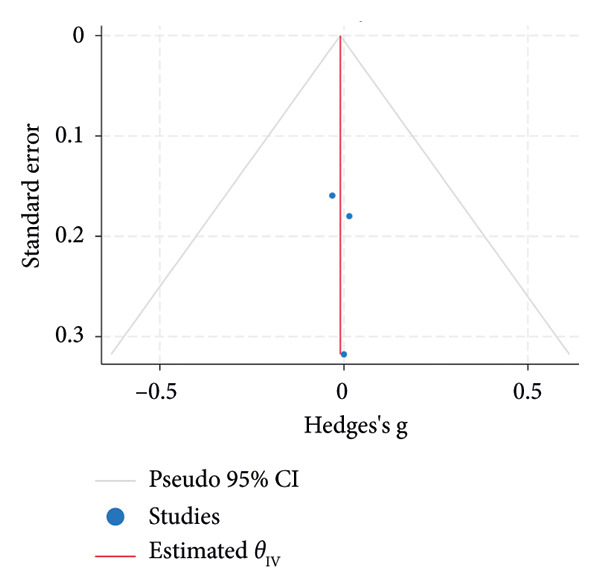
(h)

**Figure:**
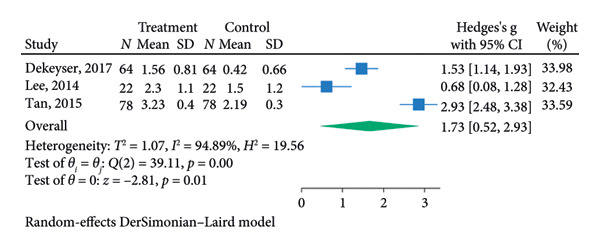
(i)

**Figure:**
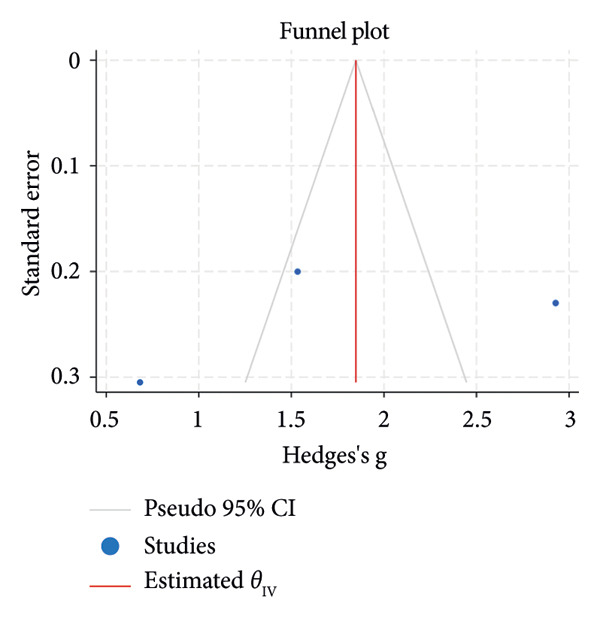
(j)

The quality of evidence supporting IOP reduction is moderate to high for STL and medication treatment, with the strength of findings supported by multiple RCTs and significant effects across different time points. The meta‐analysis found significant IOP reduction at various follow‐up times for all groups, with strong effect sizes (SMD) and confidence intervals that indicate consistent effectiveness over short‐term (1 week) to long‐term (52 weeks) periods. With the high heterogeneity (*I* ^2^ values often above 70%) in the SLT and medication groups, it suggests possible variation in study results that might weaken the overall confidence in the findings. On the other hand, the strength of evidence for combination treatment is low due to the small number of RCTs included in the meta‐analyses. Additional well‐designed RCTs, particularly for combination therapy, would strengthen the evidence and address some of the observed variability in results.

### 3.4. Number of Medications

Another meta‐analysis was performed to assess the number of medications patients were using at certain follow‐up time points. This is an important outcome in the clinical context because it greatly affects the adherence to treatment, the burden of treatment patients experience, the cost of medications, and the QoL of patients. The chronic, progressive nature of glaucoma is associated with a greater number of medications to delay the deterioration of glaucomatous optic neuropathy and visual field defect.

At 26 weeks, the number of medications for the combination therapy was reported in three RCTs. References [25, 38] did not include meta‐analysis due to the lack of information on the number of eyes allocated to each treatment [38]. The medication group did not have a significant pooled change in number of medication at the 6 months follow‐up (SMD = −0.01, 95% CI = [−0.23, 0.21], *p* = 0.93, *I* ^2^ = 0.00%, Figure 3(g)). At 6 months, the pooled results revealed that the combination treatment significantly reduced the mean number of medications used by 0.78 with no heterogeneity (SMD = 0.78, 95% CI = [0.56, 1.01], *p* < 0.01, *I* ^2^ = 0.00%; Figure 3(i)). Both funnel plots revealed moderate symmetry in the three studies (Figures 3(h) and 3(j)). A significantly lower medication use in the combination group than the medication group was reported at 6 [25, 31, 36, 38], 12 [25, 38], and 18 [38] months. The mean number of medications was significantly lower for the SLT group compared to the medication group at all time points during the 5‐year follow‐up [30]. After 1 year of SLT treatment, 77.55% of the patients who received combination treatment did not require any medication for IOP management [25].

The meta‐analyses and funnel plots indicate that the strength of evidence for the number of medications outcome across all three treatment groups is moderate, primarily due to the limited number of RCTs available. This small sample of studies limits the robustness of the findings and highlights the need for additional high‐quality studies to strengthen the evidence base. Expanding the pool of RCTs would provide greater statistical power and improve the reliability of conclusions regarding the comparative effectiveness of treatments in reducing medication burden.

### 3.5. Quality of Life

QoL for glaucoma patients is significantly impacted by the deterioration of vision and the burden of treatment. Therefore, it is important to understand how the QoL differs between the different glaucoma treatments. There are a total of five RCTs conducted QoL analyses comparing either SLT [23, 26, 27, 34] or combination therapy [31] to medication. These five studies used different QoL scales with one study using multiple scales, which include Glaucoma Outcomes Assessment Tool (GOAT), EuroQol‐5D, Glaucoma Utility Index, Glaucoma Quality of Life15, and WHO visual functioning questionnaire (WHO/PBD‐VF20). There was insufficient QoL data to perform a meta‐analysis due to the usage of various QoL scales.

Ang et al. reported that both SLT and medication treatment groups had a significant health‐related quality of life (HRQoL) improvement in the Glaucoma Outcomes Assessment Tool (GOAT) at 12 and 24 months. For the social wellbeing subdomain of the GOAT, SLT had a significantly better quality of life than patients using medication only at 24 months [23]. At 36 months, the SLT treatment group had a nonsignificant, higher QALYs than the medication group, but it was a nonsignificant difference [27]. At 72 months, there was no significant score difference in HRQoL between the SLT and the medication group for the EuroQol‐5D, Glaucoma Utility Index, and Glaucoma Quality of Life15 measurement scales [26]. But the GSS scale reported a significantly lower score in the medication group than the SLT group at 72 months [26]. Philippin et al. observed similar vision‐related QoL between the SLT and medication treatment groups [34]. The combination group had a statistically nonsignificant higher quality of life score at 6 months compared to baseline [31]. Furthermore, the changes in QoL over 6 months were similar between the combination and medication intervention groups [26].

The four studies comparing SLT to medical therapy demonstrated a moderate strength of quality based on their risk of bias assessments. These four studies found that the QoL of SLT is noninferior to medication treatment. However, with only one study investigating QoL outcomes in the context of combination therapy, there is a need for additional research to further explore and validate the QoL implications of using SLT as an adjunctive treatment with medical therapy. This additional research could help determine the broader applicability of SLT as an alternative or complement to traditional glaucoma therapies in clinical practice.

### 3.6. AOEs

The definition for AOEs differed between studies. Ang et al. recorded the presence of ocular surface disease, which is a type of AOEs that was linked to SLT or medication treatment [23]. Christie et al. reported a list of treatment‐emergent adverse events that include corneal disorder, corneal degeneration, anterior chamber flare, iritis, and many other ocular diseases [24]. Nagar et al. reported transient AOEs as discomfort or pain, uveitis, and IOP spike [33]. De Keyser et al. defined AOEs as anterior segment conditions [25]. AOEs were described as unfavorable medical occurrence, but did not necessarily result from the treatment (e.g., Gazzard et al.) [26, 27]. Overall, the general AOE definition of all these studies is an unfavorable ocular disease that occurred during the trial that may or may not have been due to the glaucoma treatment.

Three studies found that the SLT intervention had fewer AOEs compared to medication, but did not perform a statistical analysis [23, 24, 27]. Nagar et al. reported that patients treated with SLT experienced more AOEs, but was not statistically significant compared to medication [33]. Another study indicated that the SLT group was not significantly better compared to the medication group based on this outcome [34]. In contrast, De Keyser et al. observed that the SLT treatment had significantly fewer AOEs than the medication treatment [25, 26].

Because of the limited data on AOEs and the lack of specific common AOEs, the strength of evidence on the difference of AOEs between SLT, combination, and medication treatment is inconclusive. To strengthen the evidence base and facilitate a more coherent conclusion, future studies should adopt a standardized definition of AOEs. This consistency will improve the comparability of findings across studies and contribute to a more unified understanding of AOEs.

## 4. Discussion

### 4.1. Key Findings

Regarding IOP reduction, the meta‐analyses achieved significant IOP reductions in all three treatment groups for the subgroup analysis at week 26 with the greatest and least amount of IOP drop in the SLT and the combination group, respectively. A greater IOP reduction was found in the SLT group compared to the medication group for weeks 24–26 and 52, which may indicate that SLT treatment has a greater long‐term IOP reduction than medication. For number of medications, the meta‐analysis for SLT treatment showed a significant decrease in medication usage for patients at 26 weeks of follow‐up, whereas patients who received the medication treatment did not have any significant changes to their number of medications at the same follow‐up time. Furthermore, similar QoL was experienced by patients in the SLT and medication treatments. Concerning ocular adverse events, majority of the studies had fewer ocular incidents from SLT than medication. Based on all these key facets of glaucoma patients, this comprehensive systematic review and meta‐analysis suggest the noninferiority of SLT to medication treatment as both the first‐line intervention and an adjuvant treatment.

A major advantage of SLT over medical therapy alone is that SLT treatment requires further minimal action from patients, other than follow‐up appointments to monitor complications or repeat SLT treatments. As with many other chronic diseases, adherence to medical therapy can be poor in OAG patients due to vision difficulty, lack of income, or poor general health [39–41]. This can be further exacerbated, as some studies found that patients may struggle to appropriately applying topical glaucoma medications, with one specifically finding that up to a third of patients missed their eye when applying drops [42]. SLT provides a safe solution to decrease or delay patient burden in glaucoma management while maintaining comparable QoL to medication.

### 4.2. Comparison of Included RCTs

Concerning the IOP reduction outcome, the high heterogeneity could be attributed to study design differences in terms of medication usage before starting the assigned treatment, the types of SLT treatments, and the types of medication prescribed. There were six RCTs that had naïve patients [23, 26, 27, 30, 32, 35], five that had a washout period ranging from 4 to 6 weeks [24, 28, 29, 33, 34], two that kept patients on prior medication [25, 31], two that had no washout period [36, 37], and one that had no information on prior treatment [38]. The different management of medications prior to the RCT can greatly affect the IOP outcome because it has been noted that medication has a prolonged IOP reduction effect. After a 6‐week washout period, Lim et al. found that 75.3% of patients, who received prolonged monotherapy of prostaglandin, had an IOP ≤ 21 mmHg, demonstrating the lasting effects of long‐term medication use [43]. Furthermore, most studies performed 360° SLT, except Ang et al., who solely used 180° SLT [23], Nagar et al. [33] used a variety of 90° SLT, 180° SLT, and 360° SLT [33], Nagar et al. [32] did not report SLT degrees [32], and Tufan et al. used 180° SLT and 360° SLT [37], which could contribute to greater heterogeneity in study design. Majority of the RCTs used a combination of different medications including PGA, BBs, AAs, and CAIs [14, 25–27, 30, 36, 38]. Some studies solely used PGAs [23, 24, 29, 32, 33, 35], while others prescribed only BBs [34, 37].

### 4.3. Study Population

Most studies did not report participants’ level of OAG severity, while some reported mild‐moderate glaucoma status and one study with predominantly advanced glaucoma. There were RCTs that excluded patients with IOP greater than 30 mmHg. Overall, it is difficult to conclude whether our analyses would be applicable to certain glaucoma stages with such a great variety in participants’ glaucoma severity. Philippin et al. recommended that SLT would be a crucial treatment option for patients with advanced glaucoma, especially in low‐income and middle‐income regions [34]. This systematic review included broad spectrum of countries, resulting in a diverse group of participants from various ethnic, cultural, and socioeconomic backgrounds.

### 4.4. Limitations and Strengths

There were some limitations in this review. First, the heterogeneity in study design, measurement scale, population group, follow‐up time, and treatment design were evident in the included studies. Second, the lack of reported OAG severity made it impossible to evaluate the difference in treatment effect between medication and SLT for levels of OAG severity.

An important strength of this review is that its inclusion of the most up‐to‐date literature search, until January 2024, and the largest sample size to date, comprising 2412 patients. Other recent studies from Zhang et al. [16] included 2024 patients and Chavez et al. [15] included 1706 patients [15, 16]. While prior reviews also included time‐stratified outcomes, this review was able to include more granular time points and larger sample sizes at these time points as compared with prior review by Chavez et al. [15]. Furthermore, this review included a combination SLT plus mediation treatment arm and found that SLT both alone and in conjunction with medications can reduce topical medication use at 6 months.

## 5. Conclusion

This systematic review and meta‐analysis found that long‐term IOP reduction was greater in the SLT treatment compared to medication. The SLT treatment significantly reduced the number of medications used to participants, whereas no significant change in the number of medications used was found for those in the medication group. Lastly, QoL was similar between the two treatments.

## Conflicts of Interest

The authors declare no conflicts of interest.

## Funding

No funding was obtained for this study.

## Data Availability

There are no additional data available.
